# History of Yellow Fever

**Published:** 1909-10

**Authors:** 


History of Yellow Fever. By George Augustin, M. D., Assistant Secretary Louisiana State Medical Society, etc.; Author of "Romances of New Orleans" and other Creole stories, and numerous other articles, medical, historical and statistical. New Orleans. Published for the author by Searcy & Pfaff, 724 Perdido Street, of whom the book may be ordered. Cloth; 1194 pages; price $6.00.

This work is monumental and will live. Now that yellow fever is a thing of the past, at least in epidemic form, this book is timely and will stand as a monument to its memory. In it Dr. Augustin gives us all that is known of the disease, from the first observed cases away back in the early centuries of our era, with a record, more or less necessarily incomplete, of all the epidemics, and closes with a spirited and well-written account of the great discovery of the true cause of its spread. As to the ultimate cause, supposed to be a "soluble toxin" in the blood, which to date has defied the filter and the microscope; and how, when or where the first cases occurred no man knows or probably will ever know. Sufficient it is that sanitary science can now suppress and prevent its spread. Every health department should possess this truly wonderful and valuable work. D.

